# Flavonoids Induce the Synthesis and Secretion of Neurotrophic Factors in Cultured Rat Astrocytes: A Signaling Response Mediated by Estrogen Receptor

**DOI:** 10.1155/2013/127075

**Published:** 2013-06-25

**Authors:** Sherry L. Xu, Cathy W. C. Bi, Roy C. Y. Choi, Kevin Y. Zhu, Abudureyimu Miernisha, Tina T. X. Dong, Karl W. K. Tsim

**Affiliations:** Division of Life Science and Center for Chinese Medicine, The Hong Kong University of Science and Technology, Clear Water Bay, Kowloon, Hong Kong

## Abstract

Neurotrophic factors are playing vital roles in survival, growth, and function of neurons. Regulation of neurotrophic factors in the brain has been considered as one of the targets in developing drug or therapy against neuronal disorders. Flavonoids, a family of multifunctional natural compounds, are well known for their neuronal beneficial effects. Here, the effects of flavonoids on regulating neurotrophic factors were analyzed in cultured rat astrocytes. Astrocyte is a major secreting source of neurotrophic factors in the brain. Thirty-three flavonoids were screened in the cultures, and calycosin, isorhamnetin, luteolin, and genistein were identified to be highly active in inducing the synthesis and secretion of neurotrophic factors, including nerve growth factor (NGF), glial-derived neurotrophic factor (GDNF), and brain-derived neurotrophic factor (BDNF). The inductions were in time- and dose-dependent manners. In cultured astrocytes, the phosphorylation of estrogen receptor was triggered by application of flavonoids. The phosphorylation was blocked by an inhibitor of estrogen receptor, which in parallel reduced the flavonoid-induced expression of neurotrophic factors. The results proposed the role of flavonoids in protecting brain diseases, and therefore these flavonoids could be developed for health food supplement for patients suffering from neurodegenerative diseases.

## 1. Introduction

Astrocytes are the most abundant type of glial cell in nervous system, and various brain functions have been attributed to astrocytes. During the last decade, it is recognized that the functions of astrocyte are not limited in supporting neurons, but they have a number of essential activities in the brain, including the development of central nervous system (CNS), ion homeostasis, uptake of neurotransmitters, maintenance of the blood-brain barrier (BBB), and modulation of CNS immune system, as well as the synthesis of neurotrophic factors [1]. Neurotrophic factors are a group of proteins mainly synthesized and secreted by neurons and astrocytes [2, 3]: these factors are playing vital roles in maintaining the survival, growth, differentiation, and normal functions of neurons [4, 5]. Nerve growth factor (NGF) is one of the key neurotrophic factors of neurite outgrowth during development. Many diseases of nervous system are associated with NGF insufficiency, especially neurodegenerative diseases [6], for example, depression [7] and Alzheimer's disease [8]. The expressions of glial cell-derived neurotrophic factor (GDNF) and brain-derived neurotrophic factor (BDNF) are also regulated by stress-related mood disorder and depression. The amount and effectiveness of the neurotrophic factors in the brain were found to be decreased during the process of aging, and the decrease was robust in the pathological condition of Parkinson's and Alzheimer's diseases [9, 10]. Much attention has been attracted to the correlation between neurotrophic factors and neurodegenerative diseases and depression. The upregulation of NGF, BDNF, GDNF, and other neurotrophic factors is considered for treatment of depression and neurodegenerative diseases [11]. In animal model of Parkinson's disease, the delivery of GDNF gene to damaged nigrostriatal system could alleviate the symptoms in rats, which therefore implied a potential clinical use of GDNF for human [12]. The expression of BDNF is closely related to estrogen: the activation of estrogen receptor (ER) could potentially lead to production of BDNF [13]. Flavonoids, also known as phytoestrogen, have been reported to have neuronal beneficial effects including neuroprotection against neurotoxin stress, promotion of memory, learning and cognitive functions [14, 15]. Previously, it has been stated that flavonoids could protect the neurons against cell toxicity induced by oxidative stress [16] and by aggregated *β*-amyloid [17]: these stress inducers are considered as the causes of Alzheimer's disease. In addition, flavonoids were also shown to significantly potentiate NGF-induced neurite outgrowth [18], as demonstrated in cultured PC12 cells. Having a close resemblance to estrogen, the roles of various flavonoids in inducing the expression of neurotrophic factors could be an interesting question. Here, we are testing the hypothesis that the regulation of NGF, BDNF, and GDNF in cultured astrocytes could be triggered by flavonoids, and this effect could be mediated by a signaling of ER.

## 2. Materials and Methods

### 2.1. Materials

Calycosin and other flavonoids were purchased from the National Institute for the Control of Pharmaceutical Biology Products (NICPBP; Beijing, China), Sigma (St. Louis, MO) Wakojunyaku (Osaka, Japan), or Kunming Institute of Botany, Chinese Academy of Science (Kunming, China). All of them were at over 98% purity. The flavonoids were solubilized in dimethylsulfoxide (DMSO) to give stock solution at a series of concentration from 25 to 100 mM, stored at −20°C. 

### 2.2. Cell Culture and Flavonoid Treatment

Primary cultured rat astrocyte was isolated from 1-day-old neonatal rat as described previously [19] with little modification. Cells were maintained in Minimum Essential Medium (MEM) supplemented with 10% horse serum, 100 U/mL penicillin, and 100 *μ*g/mL streptomycin in a humidified CO_2_ (5%) incubator at 37°C. Fresh medium was supplied every three days. All culture reagents were purchased from Invitrogen Technologies (Carlsbad, CA). During the treatment with flavonoids, cultured astrocyte cells were serum starved for 3 days in MEM supplemented with 0.5% fetal horse serum and penicillin-streptomycin, which were then treated with the flavonoids and/or other reagents for 48 hours. 

### 2.3. Real-Time Quantitative PCR

Total RNA was isolated from cell cultures by RNAzol RT reagent according to the manufacture's instruction (Molecular Research Center, Cincinnati, OH). The purities of the RNAs were detected by UV absorbance at 260 nm. Total RNA was used to do the reverse transcription with Moloney murine leukemia virus (MMLV) reverse transcriptase according to the protocol provided by manufacturer. Real-time PCR was performed by using Roche SYBR FAST qPCR Master Mix and Rox reference dye, according to manufacturer's instruction (Roche Woburn, MA). The SYBR green signal was detected by Mx3000P multiplex quantitative PCR machine. The primers used for PCR were 5′-CAC TCT GAG GTG CAT AGC GTA ATG TC-3′ and 5′-CTG TGA GTC CTG TTG AAG GAG ATT GTA C-3′ for NGF (XP_001067130.2, 374 bp); 5′-GAG CTG AGC GTG TGT GAC AGT ATT AG-3′ and 5′-ATT GGG TAGT TCG GCA TTG CGA GTT C-3′ for BDNF (BC087634, 229 bp); 5′-GCG CTG ACC AGT GAC TCC AAT ATG-3′ and 5′-CGC TTC ACA GGA ACC GCT ACA ATAT C-3′ for GDNF (AF497634, 318 bp); 5′-AAC GGA TTT GGC CGT ATT GG-3′ and 5′-CTT CCC GTT CAG CTC TGG G-3′ for GAPDH ([20], 516 bp).

### 2.4. Measurement of Secretion of NGF, BDNF, and GDNF

To measure the protein level of secreted neurotrophic factors in primary cultured rat astrocyte, the method of enzyme-linked immunosorbent assay (ELISA) was employed. Rat astrocytes were plated in a 12-well plate in MEM supplemented with 10% horse serum, 100 U/mL penicillin, and 100 *μ*g/mL. When the confluence of cells was higher than 80%, the medium was changed into MEM with 0.5% horse serum, 100 U/mL penicillin, and 100 *μ*g/mL streptomycin for other weeks and was changed again to get equal volume 3 hours before the drug treatment. Flavonoids and other drugs were applied to cell culture and lasted for 48 hours. Then the medium and cell culture were collected and stored in −80°C. The protein amount of neurotrophic factors in the medium was measured with the method of ELISA, and the protein concentration of the cell lysate for each sample was also measured. The ELISA assays were performed with the commercially available ELISA kits (AbFrontier, Millipore) for NGF, BDNF, and GDNF measurement according to the manufacturer's instructions. Briefly, samples were applied onto a 96-well plate precoated with anti-rat NGF, BDNF, or GDNF antibodies and incubated on at 37°C for 90 minutes. After discarding plate content, biotinylated anti-rat NGF, BDNF, or GDNF antibodies were added and incubated at 37°C for 60 minutes. After washing with PBS for four times, the avidin-biotin-peroxidase complex solution was added and incubated at 37°C for 30 minutes. Tetramethylbenzidine solution was added and incubated at 37°C for 15 minutes. The reaction was stopped with 1 M sulfuric acid and absorbance recorded at 450 nm, immediately. The values of standards and samples were corrected by subtracting the absorbance of nonspecific blinding. All samples were measured in triplicate in the same assay to minimize interassay variation.

### 2.5. Estrogen Receptor Phosphorylation

Astrocytes were seeded onto 12-well plates. When the confluence of cells in the plate reached to 90%, the culture medium was changed to MEM medium without any serum. After serum starvation for at least 5 hours, the cells were treated with drugs at different time points (e.g., 0 to 30 minutes). Then, the cells were harvested and digested with 2 X SDS-PAGE sample buffer (0.125 M Tris-Cl, pH 6.8, 4% SDS, 20% glycerol, 2% 2-mercaptoethanol, and 0.02% bromophenol blue) by shaking for 2 minutes and boiling for 15 minutes. The proteins were subjected to SDS-gel electrophoresis and blotting. The membrane containing the transferred proteins was incubated with antiphospho-ER*α*-S118 antibody (1 : 2000; Upstate, Lake Placid, NY) and antitotal ER*α* antibody (1 : 1000; Upstate) at 4°C for 12 hours. Horseradish-peroxidase- (HRP-) conjugated anti-rabbit secondary antibody (1 : 5000; Invitrogen) was then added to the membranes for 1 hour at room temperature. The secondary antibody, horseradish-peroxidase- (HRP-) conjugated anti-rabbit antibody (1 : 5000; Invitrogen) was then added to the membranes for 1 hour at room temperature. The immunecomplexes were visualized by the enhanced chemiluminescence (ECL) method (GE, Healthcare). The band intensities, recognized by the antibodies in the ECL film, in control and flavonoid-treated samples were run on the same gel and under strictly standardized ECL conditions. The bands were compared on an image analyzer, using in each case a calibration plot constructed from a parallel gel with serial dilution of one of those samples as to ensure the subsaturation of the gel exposure. Total amount of ER*α* was detected as an internal control.

### 2.6. Statistical Analysis and Other Assays

The protein concentrations were measured routinely by Bradford's method (Hercules, CA). Statistical analyses were performed using one-way ANOVA followed by Student's *t*-test. Statistically significant changes were classed as (*) where *P* < 0.05; (**) where *P* < 0.01.

## 3. Results

### 3.1. Screening for Flavonoids in Increasing the Expression of Neurotrophic Factors

Rat astrocytes were isolated and cultured. The cells were proliferated in culture for about 3 weeks, which reached maximum cell number under different plating cell numbers (See Supplementary Figure 1 Material available online at http://dx.doi.org/10.1155/2013/127075). The culture showed over 90% identity of astrocyte, that is, the specific staining of GFAP (Supplementary Figure  2). Thirty-three flavonoids from different subclasses were screened for their effects on secretion of neurotrophic factors on cultured astrocytes. These flavonoids are mainly derived from vegetables and Chinese herbal medicines. According to the results of cell viability assay, flavonoids were applied onto the cultures at concentrations of 10 *μ*M, at which the flavonoids induced neither cell proliferation nor cell toxicity. After incubation for 48 hours, the culture medium was harvested to perform the ELISA assay in measuring the concentrations of NGF, GDNF, and BDNF. The results were normalized by protein concentrations of cell lysates from each sample. Estrogen was shown to induce the expression and secretion of neurotrophic factors in cultured hippocampal neuron [21], and therefore 17*β*-estradiol served as the positive control. From Table 1, several flavonoids showed significant effects in upregulating secretion of NGF, GDNF, and BDNF. Alpinetin, luteolin, calycosin, genistein, and isorhamnetin were revealed in inducing the expression of NGF, GDNF, and BDNF, significantly. On the other hand, the NGF-induced flavonoids was included silybin, calycosin-7-O-glucoside, and fiestin. The GDNF-induced flavonoids were naringin, neohesperidin, apigenin, sulphureting, cardamonin, calycosin-7-O-glucoside, puerarin, galangin, and RNFG. The BDNF-induced flavonoids were very limited (Table 1).

### 3.2. Flavonoids Increase the Expression of NGF, BDNF, and GDNF

**Figure 1:**
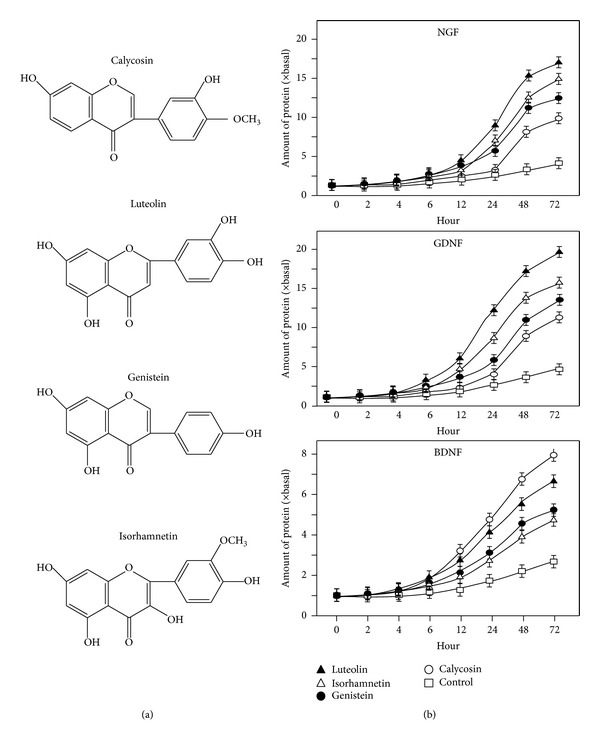
Flavonoids increase the protein expressions of neurotrophic factors in time-dependent manner. (a) The chemical structures of the most effective flavonoids, calycosin, luteolin, genistein, and isorhamnetin, in inducing neurotrophic factors in cultured astrocytes. (b) Calycosin, isorhamnetin, luteolin and genistein at the concentration of 10 *μ*M were applied to cultured astrocytes. Conditional medium was collected at different time points (0–72 hours), and the concentrations of neurotrophic factors were measured by ELISA kits. Values are expressed as the fold of change (× basal) against the control (no treatment at various time points; set as 1) and in Mean ± SEM, *n* = 3.

**Figure 2:**
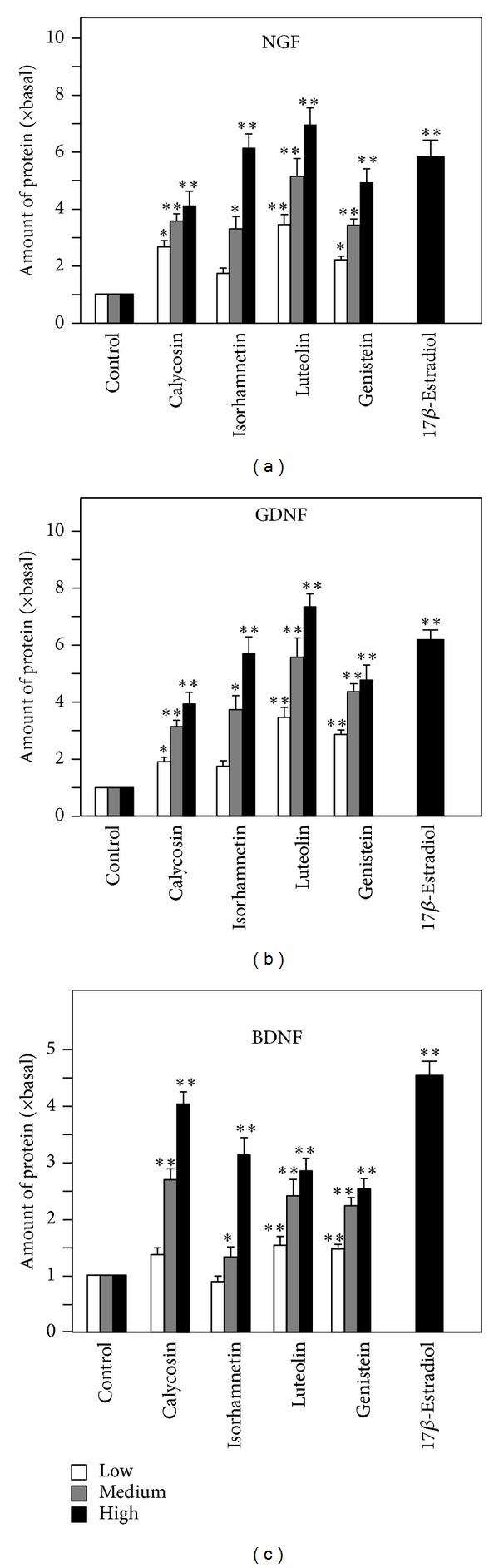
Flavonoids increase the expression of NGF, GDNF, and BDNF in cultured astrocytes in dose-dependent manner. The flavonoids calycosin, isorhamnetin, luteolin, and genistein (1, 3, 10 *μ*M) were applied to cultured astrocytes for 48 hours. 17*β*-estradiol at 100 nM served as the positive control. The protein levels of NGF, GDNF, and BDNF in the conditional medium were measured by ELISA. Values are expressed as the fold of change (× basal) against the control (no treatment; set as 1) and in Mean ± SEM, *n* = 4, each with triplicate samples. ∗∗ where *P* < 0.01 compared to the control.

**Figure 3:**
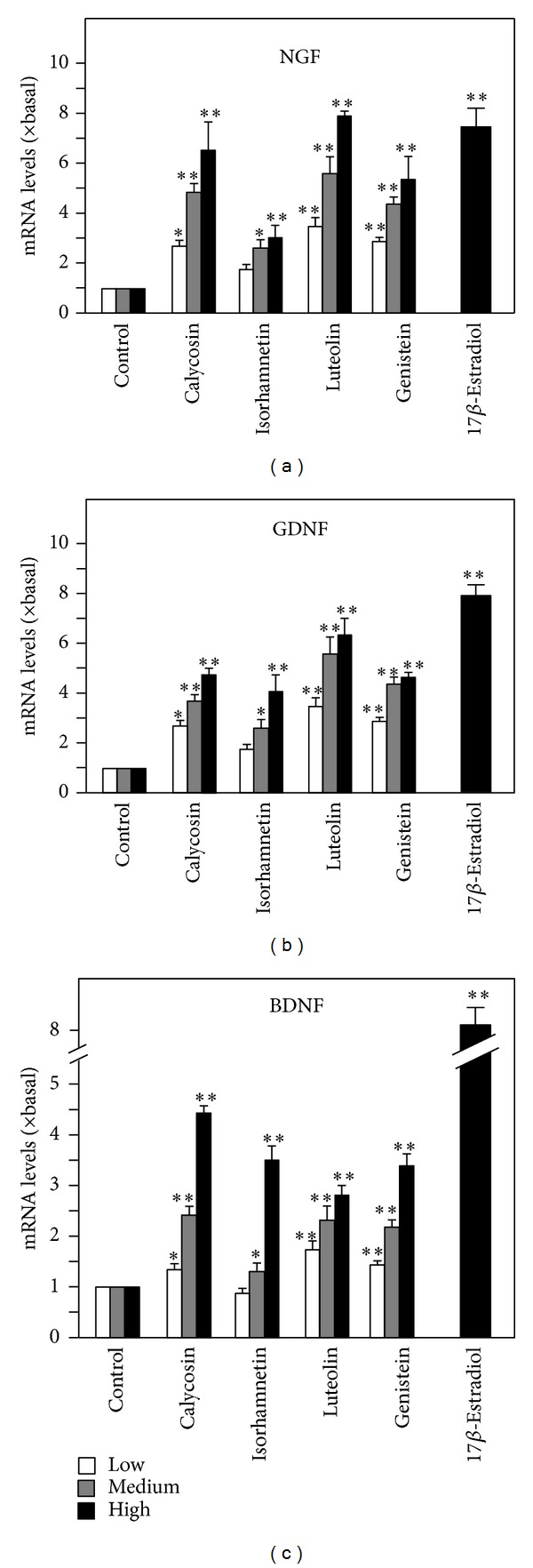
Flavonoids increase the mRNA levels of NGF, GDNF, and BDNF in cultured astrocytes in dose-dependent manner. The flavonoids calycosin, isorhamnetin, luteolin and genistein (1, 3, 10 *μ*M) were applied to cultured astrocytes for 48 hours. 17*β*-estradiol at 100 nM served as the positive control. The mRNA levels of NGF, GDNF, and BDNF were measured by real-time quantitative PCR. Values are expressed as the fold of change (× Basal) against the control (no treatment; set as 1), and in Mean ± SEM, *n* = 4, each with triplicate samples. ∗∗ where *P* < 0.01 compared to the control.

Among these effective flavonoids, luteolin from Lonicerae Japonicae Flos, isorhamnetin from Ginkgo Folium, genistein from Soybean, and calycosin from Astragali Radix (Figure 1(a)) showed the most promising effects by increasing the secretion of the three neurotrophic factors, which therefore were selected for further elucidation. These flavonoids were applied onto cultured astrocytes for different time points up to 72 hours, and the amount of neurotrophic factors in the conditioned medium was determined by ELISA. The flavonoids induced the amounts of NGF, GDNF and BDNF in the conditioned medium in a time-dependent manner: the increase was significantly at 12 hours after the treatment (Figure 1(b)). The induction was more robust in cases of NGF and GDNF; this induction was higher than that of BDNF by 2 folds. Besides, the flavonoid-induced expression of neurotrophic factors was also in a dose-dependent manner (Figure 2). In most cases, the lowest concentration of flavonoids (i.e., at 1 *μ*M) was able to induce the neurotrophic factor expressions (Figure 2). Here, the induction by estrogen was serving a positive control. The mRNA expressions of NGF, GDNF, and BDNF were revealed in cultured astrocytes under the treatment of different flavonoids, including luteolin, isorhamnetin, genistein, and calycosin. The treatment was at 48 hours, and then the total RNA was subjected to real-time quantitative PCR. Similar to the protein expression, the mRNAs encoding neurotrophic factors were induced by the flavonoids in a dose-dependent manner (Figure 3). The magnitude of induction was very similar in the scenario of protein expression, which suggested the transcriptional regulation of these neurotrophic factors could be a major step in regulation by flavonoids. Among these flavonoids, luteolin at 10 *μ*M showed the most promising effects in increasing the mRNA levels of NGF and GDNF to more than 7 folds, while calycosin at 10 *μ*M showed most promising effect in increasing the mRNA level of BDNF to more than 4 folds (Figure 3). 17*β*-Estradiol served as the positive control.

### 3.3. The Flavonoid-Induced Expression of Neurotrophic Factors Is Mediated by Estrogen Receptor

**Figure 4:**
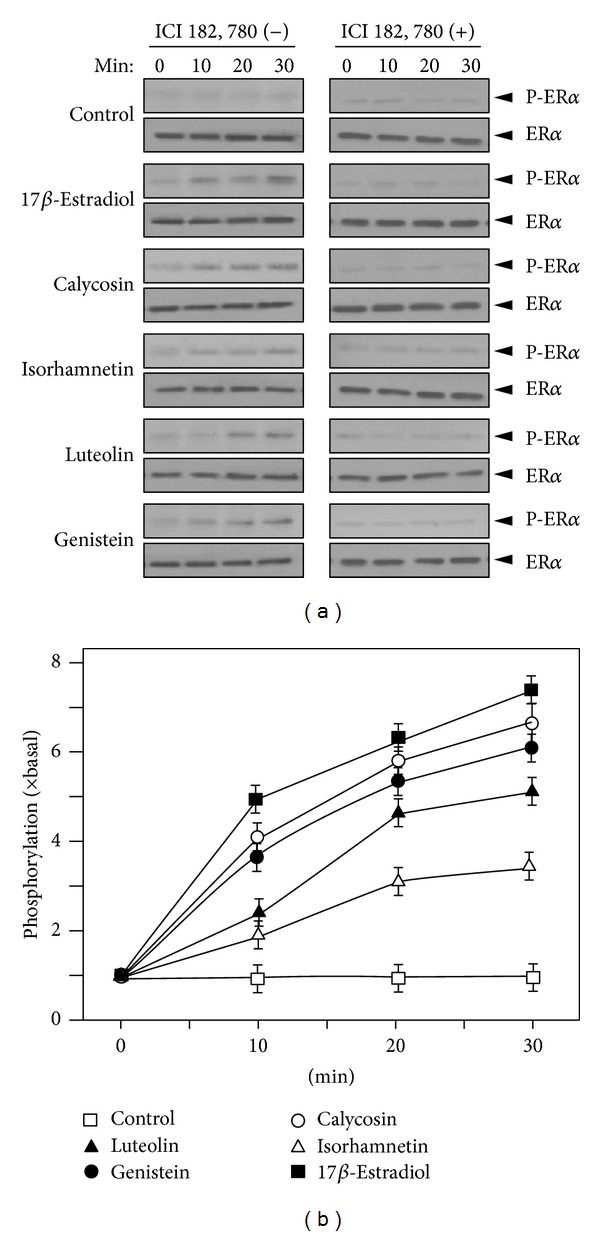
Flavonoids induce the phosphorylation of estrogen receptor. (a) Cultured astrocytes were serum starved for 3 hours with or without the pretreatment of ICI 182, 780 for another 3 hours. Then calycosin, isorhamnetin, luteolin, and genistein at 10 *μ*M were applied onto the cell cultures for a different time. 17*β*-Estradiol (10 nM) served as the positive control. Total ER*α* and S118 phosphorylated ER*α* (both at ~66 kDa) were revealed by using specific antibodies. (b) Quantification plot for the phosphorylation level of ER induced by flavonoids. Data are Means ± SEM, *n* = 3, each with triplicate samples.

**Figure 5:**
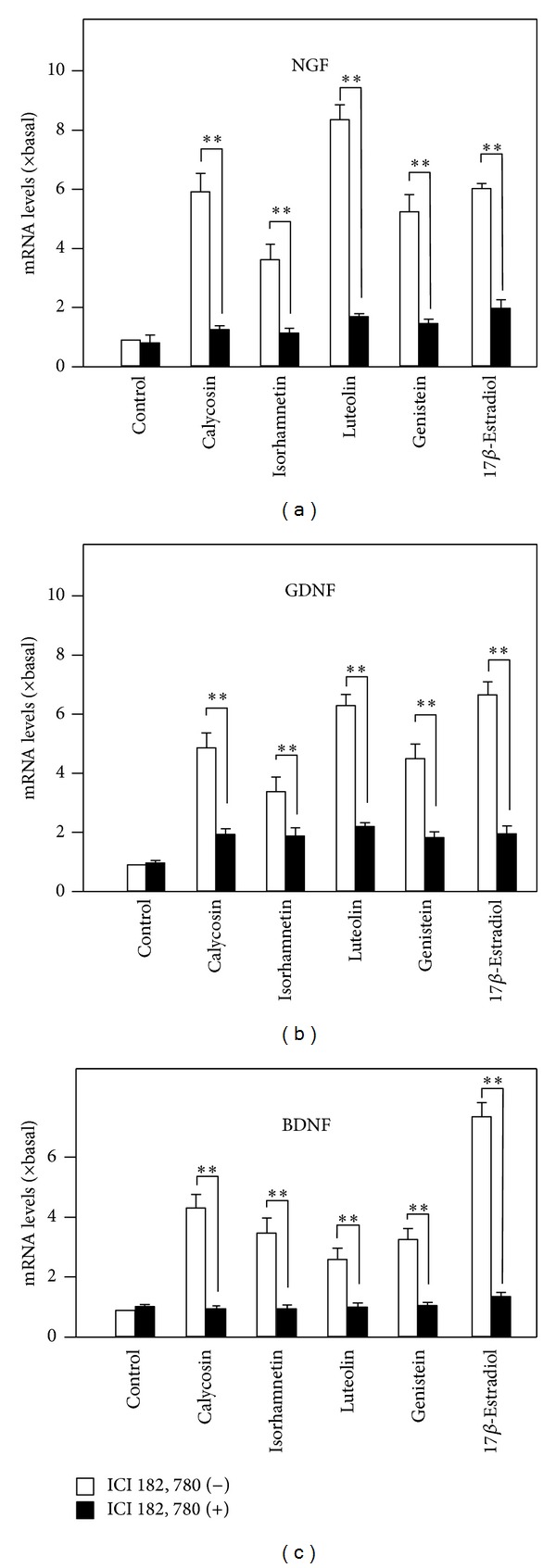
The flavonoid-induced expressions of neurotrophic factors are blocked by inhibitor of estrogen receptor. Cultured astrocytes were serum starved for 3 hours with or without the pretreatment of ICI 182, 780 for another 3 hours, as in Figure 4. Astrocytes were pretreated with ICI 182, 780 (1 *μ*M) for 3 hours and then treated with flavonoids for 48 hours. The mRNA expression levels of neurotrophic factors were analyzed. Values are expressed as the fold of change (× basal) against the control (no treatment; set as 1), and in Mean ± SEM, *n* = 4, ∗∗ where *P* < 0.01.

Since calycosin, isorhamnetin, luteolin, and genistein showed significant effects in modulating mRNA expression and protein secretion of NGF, GDNF, and BDNF, the potential molecular mechanism was elucidated. As mentioned before, estrogen signaling pathway was considered to be closely related to the expression of neurotrophic factors in the brain [21, 22]. The activation of estrogen-mediated transcription requires the phosphorylation of ER, either *α* or *β* forms [23]. Different flavonoids, when applied in cultured MCF-7 cells, triggered the estrogenic pathway by phosphorylating ER*α* at S118 position, as well as the estrogen responsive element [16]. Here, the effects of these flavonoids in activating ER*α* were firstly determined in astrocytes. The flavonoids were applied onto the cultures at different time points. From Figures 4(a) and 4(b), calycosin, isorhamnetin, luteolin, and genistein induced ER*α* phosphorylation at S118, significantly. The phosphorylation was observed from 10 minutes after treatment and lasted for at least 30 minutes at maximal phosphorylation (Figures 4(a) and 4(b)). The phosphorylation induced by flavonoids could be fully blocked by the ER antagonist ICI 182, 780 (Figure 4(a)). ICI 182, 780 was used to further investigate the relation between flavonoids induced neurotrophic factors expression and ER-dependent signaling pathway. ICI 182, 780 was applied onto astrocytes 3 hours before the flavonoid treatments, and the mRNA levels of NGF, BDNF, and GDNF were measured. With the pretreatment of ICI 182, 780, the mRNA levels of the neurotrophic factors, increased by calycosin, isorhamnetin, luteolin, and genistein, were blocked close to the basal level. 17*β*-Estradiol served as the positive control (Figure 5). These results suggested that the activities of calycosin, isorhamnetin, luteolin, and genistein in increasing the expression of NGF, BDNF, and GDNF were achieved through ER-mediated signaling pathway.

## 4. Discussion

Astrocyte is the most dominant and functional type of neuroglial cell; however, the study about relations between flavonoid and astrocyte is very limited. At the same time, neurotrophic factor has been studied as an important direction to alleviate neurodegenerative disease and depression. So far, most of these works were carried out in animal models [9–11]. Here, the roles of flavonoids in regulating neurotrophic factors were investigated in cultured astrocytes. The effects of calycosin, isorhamnetin, luteolin, and genistein in enhancing neurotrophic factor expressions were closely related to an ER-dependent pathway. Even though there was no direct evidence showing that estrogen could trigger the neurotrophic factor expression in astrocytes; studies had demonstrated that estrogen possessed activities in astrocytes. For example, the expression levels of glutamate transporters were increased by the applied estrogen in cultured astrocytes [24], and estrogen reduced lipopolysaccharide-induced expression of tumor necrosis factor-*α* and interleukin-18 in midbrain astrocytes [25]. On the other hand, estrogen could modulate the expression of neurotrophic factors in neuronal cells [21, 26, 27], which also enhanced mRNA expression of BDNF by phosphorylating CREB in rat hippocampus [28]. In a study of ER-dependent pathway, the activated ER dimer was shown to bind onto a DNA segment of upstream of BDNF promoter: the binding promoted the mRNA expression of BDNF in hippocampal neuron [29]. 

Estrogen could be synthesized directly in nervous system [30]; meanwhile, ER (both *α* and *β* form) and GPR30 are both widely distributed in the nervous system [31, 32]. Estrogen affects synaptogenesis and morphological plasticity within the brain by enhancing the density of dendritic spines [33, 34] and promoting subsequent synapse formation [35, 36]. In line to this notion, a study showed that ER*α* played a critical role in estrogen-induced glutamatergic synapse formation, including the expressions of presynaptic vesicular glutamate transporter protein (vGlut1) and postsynaptic NMDA receptor (NR1 subunit) [37]. Flavonoids are well-known phytoestrogens with multiple activities in different systems [38, 39]. Much attention has been focused on the neuronal beneficial effects of flavonoids, including the neuroprotection against neurotoxin stress [16] as well as the promotion of memory and learning and cognitive functions [15]. Previous studies in cultured neurons showed that flavonoids possessed the abilities of antioxidation and inhibiting A*β*-induced cytotoxicity [16, 17]. Many flavonoids showed estrogenic effects by directly inducing the ER phosphorylation [40–43]. Here, the correlation between estrogen signaling pathway and neuronal beneficial effects of flavonoids was innovatively demonstrated. These results explained the mechanism of flavonoids in regulating the expression of neurotrophic factors. Being the very popular phytoestrogen, the properties of flavonoids in the brain health could be the potential candidates for drug development for different types of neurodegenerative diseases.

## Supplementary Material

Supplementary Figure 1: Growth curve of cultured astrocytes.
Supplementary Figure 2: Morphology and immunostaining of cultured rat astrocytes.

## Figures and Tables

**Table 1:** The bioactivities of flavonoids in increasing the protein levels of neurotrophic factors.

Flavonoid	NGF	GDNF	BDNF
Flavanones			
Alpinetin	+++	++	+
Hesperidin	—	+	—
Naringenin	—	—	—
Naringin	—	+++	—
Neohesperidin	—	++	—
Flavones			
Apigenin	—	++	—
Baicalein	—	—	—
Luteolin	++++	++++	++
Tangeretin	++	—	—
Wogonin	—	—	—
Aurones			
Sulfuretin	—	++	—
Dihydrochalcones			
Phloretin	—	—	—
Flavonols			
Silybin	++++	—	+
Chalcones			
Cardamonin	+	++	—
Isoflavones			
Calycosin	+++	++++	+++
Calycosin-7-O-glc	+++	++	—
Daidzein	—	—	—
Formononetin	—	—	—
Genistein	++++	++++	++
Genistin	—	—	—
Puerarin	+	+++	—
Flavones			
(−)-Catechin	—	—	+
(−)-Epicatechin	—	—	—
Flavonols			
Fisetin	+++	—	+
Galangin	—	++	—
Hyperin	—	—	—
Icariin	—	—	+
Isorhamnetin	++++	++++	+++
Kaempferol	+	+	+
Quercetin	—	+	—
RNFG	—	++	—
173-estradiol	++++	++++	+++

Percentage of increas + > 100%, ++ > 200%, +++ > 300%, ++++ > 500%.

Flavonoids at 10 *μ*M were applied to cultured rat astrocytes and maintained for 48 hours. The protein levels of NGF GDNF and BDNF were measured by ELISA. 17*β*-Estradiol at 100 nM served as the positive control. Data are Means ± SEM, *n* = 3, each with triplicate samples. The value of SEM is within 5% of the mean, which is not shown for clarity. “+” to “+++” indicate the ranking of the inductive effect on protein amount of neurotrophic factors. “—” indicates no effect, that is, below 10% increase in the tested activities.
