# Supplementary material for: Flavonoids Induce the Synthesis and Secretion of Neurotrophic Factors in Cultured Rat Astrocytes: A Signaling Response Mediated by Estrogen Receptor
Source: Evid Based Complement Alternat Med. 2013 Jun 25;2013:127075. doi: 10.1155/2013/127075 (PMC3708423; doi:10.1155/2013/127075)
Supplement: Supplementary file 1 — Supplementary Figure 1: Growth curve of cultured astrocytes. Supplementary Figure 2: Morphology and immunostaining of cultured rat astrocytes. [file 127075.f1.docx]

**Supplementary Figure 1: Growth curve of cultured astrocytes**

Astrocytes were plated in 12-well plates in the densities of 15,000, 30,000, and 60,000 cells/well. The cell numbers were measured every 3 days. Even though the growth rates are different, with the initial densities of 30,000, and 60,000/well, the cell cultures reached around 80% confluence at day 15, and almost 100% confluence at day 21. Values are in Mean ± SEM, *n* = 3.

**Supplementary Figure 2: Morphology and immunostaining of cultured rat astrocytes**

**(A):** The morphology of cultured rat astrocytes was observed under the microscopy. Bar = 10 μm.

**(B):** Cultured astrocytes were stained by anti-GFAP polyclonal antibody (shown in red; from Sigma-Aldrich dilution 1:1000), anti-NF68 polyclonal antibody (shown in green; from Sigma-Aldrich dilution 1:1000) and DAPI (shown in blue, for nucleus staining; from Sigma-Aldrich dilution 1:1000) in the present of 0.1% Triton-X100. The staining protocol was referring to [[44](#_ENREF_44)]. Bar = 20 μm.


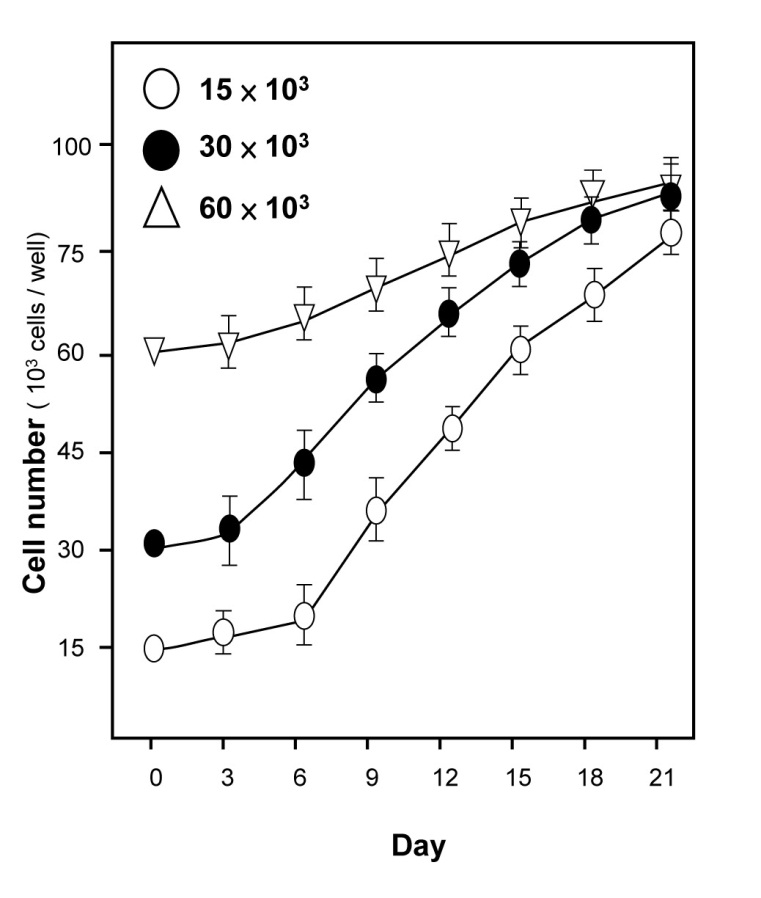


**Supplementary Figure 1**


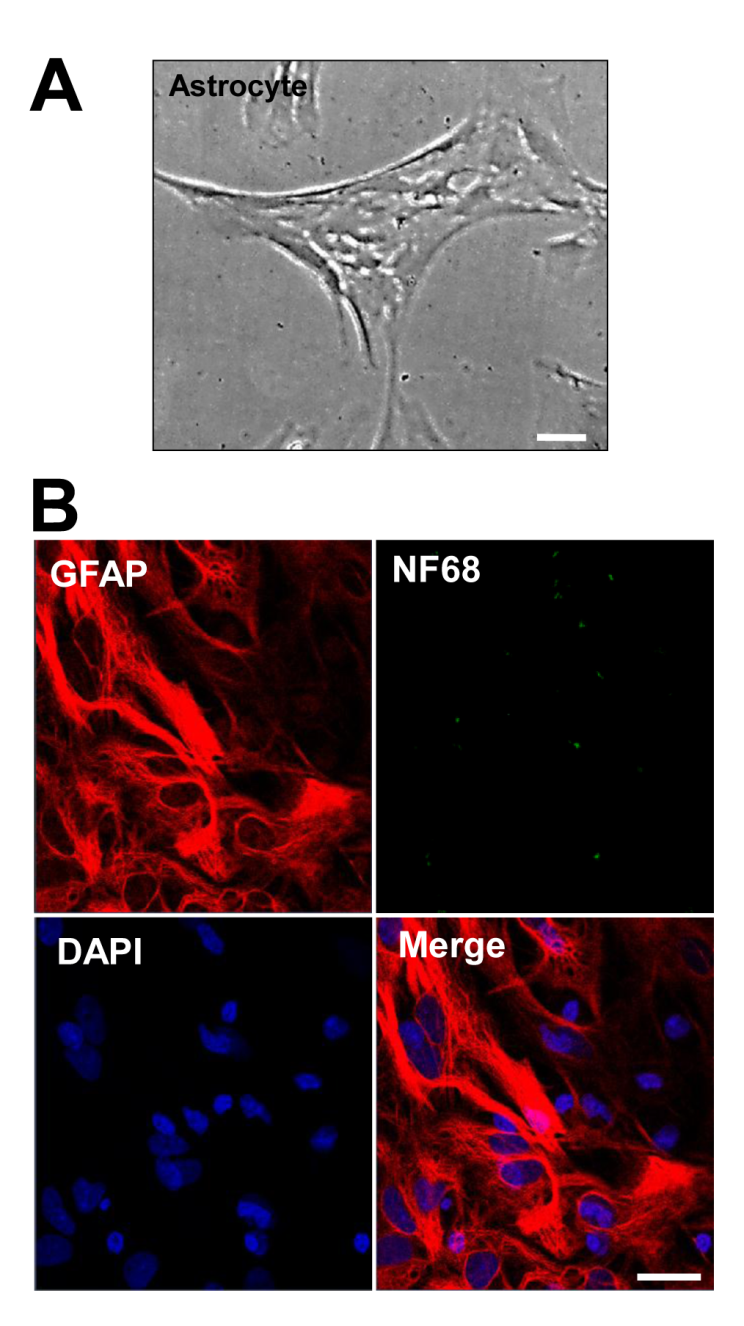


**Supplementary Figure 2**
